# Meta-Analysis of Dyslipidemia and Blood Lipid Parameters on the Risk of Primary Open-Angle Glaucoma

**DOI:** 10.1155/2022/1122994

**Published:** 2022-09-21

**Authors:** Guimei Huang, Jiayi Wang, Lei Li, Yuan Gao, Yijie Yan

**Affiliations:** ^1^Ophthalmology Department, Qionghai People's Hospital, Qionghai, 571400 Hainan, China; ^2^Ophthalmology Department, Qionghai Jianping Hospital, Qionghai, 571400 Hainan, China; ^3^Ophthalmology Department, Hainan General Hospital (Hainan Affiliated Hospital of Hainan Medical University), Haikou, 570311 Hainan, China

## Abstract

**Objective:**

We aimed to explore the effect of blood lipid parameters on the risk of primary open-angle glaucoma (POAG) by meta-analysis.

**Methods:**

The databases of PubMed, Scopus, CNKI, and Wanfang were systematically searched from inception to April 2022, and the relevant research literature was obtained, screened, and analyzed.

**Results:**

A total of 15 studies were included in this meta-analysis, including 11 reporting dyslipidemia and risk of POAG and 5 reporting specific lipid level and risk of POAG. Dyslipidemia increased the risk of POAG with an odd ratio (OR) of 1.25 (95% CI: 1.23, 1.26). Total triglyceride and total cholesterol were not related to the prevalence of POAG, but high-density lipoprotein cholesterol was significantly negatively correlated with the risk of POAG with an OR of 0.96 (95% CI: 0.94, 0.99).

**Conclusion:**

Dyslipidemia is a risk factor for POAG. Given the small sample size and significant interstudy heterogeneity, additional studies are needed to establish this conclusion.

## 1. Introduction

Glaucoma is a group of ocular conditions characterized by progressive optic nerve damage with corresponding visual field defect. Pathologically, glaucoma is characterized by the loss of retinal ganglion cells, the thinning of the retinal nerve fiber layer and the morphological change of the optic disc. Primary open-angle glaucoma (POAG) is a type of glaucoma with an open, normal-appearing anterior chamber angle and raised intraocular pressure, in the absence of other underlying diseases [1]. According to prior reports, the number of patients with glaucoma worldwide is 76 million with an incidence of 3.54% in people aged 40-80, which is expected to be increased to 112 million by 2040. The exact pathogenesis of POAG remains unclear. The risk factors identified include elevated intraocular pressure [2], advanced age [3], race [4], and a family history of glaucoma [5]. The study by Jonas et al. that first evaluated the relationship between blood lipid levels and POAG found no significant correlations [6]. A recent metastudy showed that the total triglyceride level in POAG was significantly higher than that of the control group [7]. This finding is echoed by a recent meta-analysis [8]. Nonetheless, the associations between specific blood lipid parameters and the risk of POAG remain scarcely investigated. The purpose of this study is to explore the relationship between dyslipidemia, specific blood lipid parameters, and the prevalence or risk of POAG through a meta-analysis.

## 2. Materials and Methods

### 2.1. Literature Search

The databases PubMed, Scopus, CNKI, and Wanfang were searched from inception to May 6, 2022. The search language was limited to Chinese and English. The search terms were as follows: “glaucoma, open angle” or “open angle glaucomas” and “cholesterol,” “glyceride,” “high-density cholesterol lipoprotein,” “low-density cholesterol lipoprotein,” and “blood lipid,” “lipid metabolism,” or “hypercholesterolemia, dyslipidemia,” and “odds ratio”.

### 2.2. Inclusion and Exclusion Criteria

Inclusion criteria: (i) The study purpose was to explore the effect of blood lipid level (or abnormal lipid metabolism) on the risk of POAG; (ii) Study design was cross-sectional, case-control, or cohort study; (iii) Publication language limited to Chinese or English; (iv) Study population is not limited by age, race, and gender.

Literature exclusion criteria: (i) The literature content is inconsistent or weakly relevant; (ii) Animal experiments, in vitro tests, letters, reviews, case reports, abstracts, or incomplete reports; (iii) No data available in terms of study exposure (no blood lipid level) or relevant outcomes; (iv) No original text.

### 2.3. Data Sorting

Yan completed the content extraction from eligible publications, while Huang and Wang checked independently. The contents extracted included study author name, year of publication, study type, inclusion criteria of patients, number of patients, main observation indicators, and other information.

### 2.4. Literature Quality Evaluation

The quality of the included publications was evaluated by the scale reported by Viswanathan et al. [9, 10]. The quality assessment method included 15 items involving the design, criteria for observational studies, and data analysis evaluation. It evaluated the methods of research objects selection, results and exposure measurement, and the methods of controlling confounders, potential conflicts of interest and the risk of deviation related to different designs. A score of 0 or 1 would be assigned to each item assessed, and the total score would be 15 points.

### 2.5. Statistical Analysis

All data in this study were analyzed by Stata v16.0 software. Two-sided *P* < 0.05 denoted statistically significance. The binary enumeration data was expressed as odds ratio (OR) with 95% confidence interval (95% CI). If the OR was not provided, it was calculated according to the number of events in the diseased and non-diseased population. The specific calculation equation is OR = (number of exposed persons in the diseased group/number of nonexposed persons in the diseased group)/(number of exposed persons in the nondiseased group/number of nonexposed persons in the nondiseased group). The heterogeneity test between different studies was described by the I^2^ statistic with the following equation *I*^2^ = (Q − df)/Q, in which *Q* represents the *χ*2 statistic and df denotes its degrees of freedom). I^2^ corrected by degrees of freedom ≥50% or <50% was deemed to indicate high or low inter-study heterogeneity, respectively. All studies were pooled using the fixed-effect models. The association between hyperlipidemia and glaucoma was evaluated according to the study design, and subgroup analysis was performed. Publication bias was assessed using Begg's funnel plot, along with Begg's test. The Begg's test is a rank correlation funnel plot asymmetry test method, and when *P* > 0.05, it can be considered that the funnel plot has no obvious asymmetry. When the data is less than 10 points, the funnel plot and asymmetric test methods cannot judge whether there is publication bias due to the low-test power. In this study, we do not report funnel plots with fewer than 10 points, but still report the results of Begg's test.

## 3. Results

### 3.1. Literature Search

**Figure 1:**
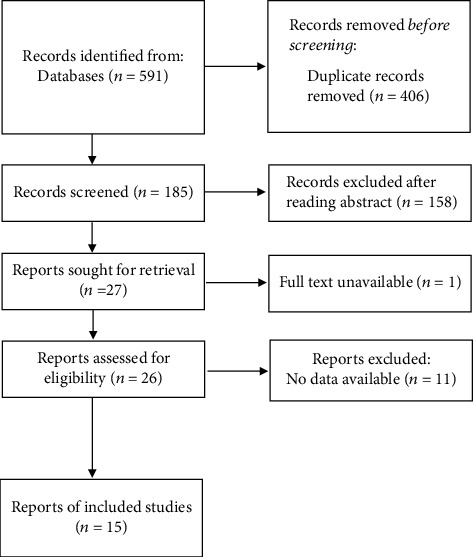
Literature screening flowchart.

The screening process of this study is shown in Figure 1. A total of 591 articles were retrieved, including 585 relevant Chinese and English documents and 6 ambiguous documents. After removing 406 duplicate documents, 158 documents were screened and excluded by reading the title and abstract. The full-texts of the remaining 27 documents were read, and 11 publications were excluded for absence of original text (*n* = 1) and data unavailability (*n* = 10). Of the 15 included literature, 11 reported abnormal lipid metabolism and risk of POAG, and 5 reported the relationship between blood lipid level and POAG.

### 3.2. Basic Information Included in the Study

The 11 literature that reported the prevalence or risk of dyslipidemia in POAG are shown in Table 1. A total of 2879714 patients, including 155928 POAG, were involved in 4 cross-sectional studies, 4 case-control studies, and 3 cohort studies. Eight studies were conducted in Asian populations (3 in Taiwan and 5 in South Korea), and 3 in European and American populations (all conducted in the United States). The five literature that reported the risk of blood lipid level are shown in Table 2. A total of 23296 patients, including 1315 POAG, were studied in 2 cross-sectional studies and 3 case-control studies in Asian populations (3 in China and 2 in South Korea). The quality of the literature was evaluated, and the results are shown in Table 3.

### 3.3. Abnormal Lipid Metabolism and Risk of POAG

**Figure 2:**
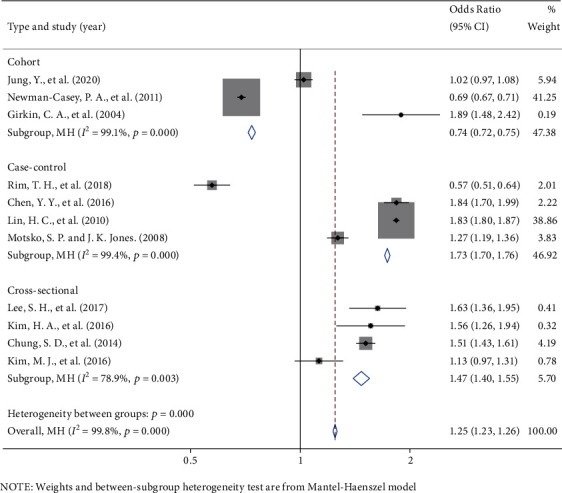
Forest chart reporting the study on the relationship between dyslipidemia and the risk of POAG.

Meta-analysis of 11 literature indicated that the OR for POAG with abnormal lipid metabolism was 1.25 (95% CI: 1.23, 1.26). Significant interstudy heterogeneity (*I*^2^ = 99.8%, *P* < 0.001) was noted and presented in Figure 2. Subgroup analysis found that both cross-sectional and case-control studies showed a positive correlation between dyslipidemia and risk of POAG with an OR of 1.47 (95% CI: 1.40, 1.55) for cross-sectional studies and 1.73 (95% CI: 1.70, 1.76) for case-control studies. Nonetheless, cohort studies showed a negative correlation between dyslipidemia and the risk of POAG with an OR of 0.74 (95% CI: 0.72, 0.75). The funnel plot (Figure S1, supplementary data) showed that the points were distributed on both sides, showing an inverted funnel shape, and *P* = 0.755 by Begg's test. Still, some studies were not within the confidence interval due to significant heterogeneity.

### 3.4. Blood Lipid Level and Risk of POAG

**Figure 3:**
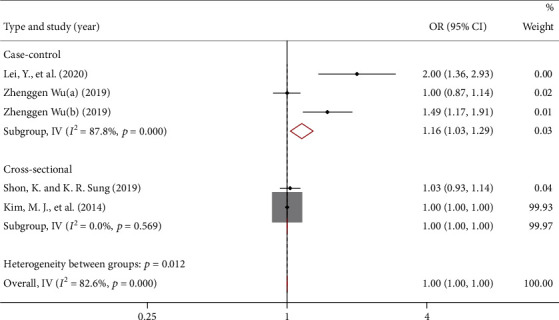
Forest map reporting the odds ratio for POAG risk by TG level.

Meta-analysis conducted on the 5 literature reporting associations between blood lipid levels and risk of POAG showed the OR for POAG by triglyceride (TG) was 1.00 (95% CI: 1.00, 1.00). There was great heterogeneity among the studies (*I*^2^ = 82.6%, *P* < 0.001), as shown in Figure 3. Subgroup analysis showed significant positive correlation between TG and risk of POAG (OR = 1.73, 95% CI: 1.70, 1.76) in case-control studies but not in cross-sectional studies. No publication bias was found by Begg's test (*P* = 0.086).

**Figure 4:**
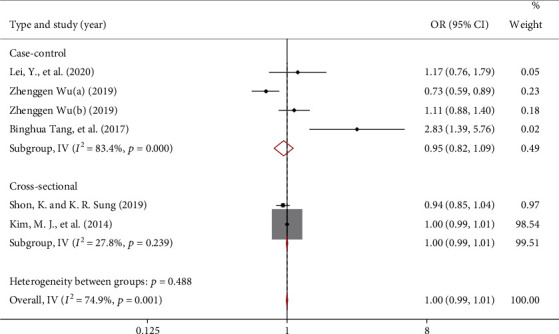
Forest map reporting the study on the association between total cholesterol and POAG risk.

**Figure 5:**
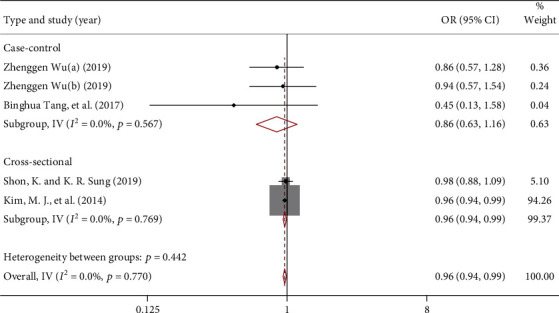
Forest map reporting the study on the association between HDLC and POAG risk.

For specific lipid levels, the meta-analysis showed that the OR for POAG risk by total cholesterol (TC) or high-density lipoprotein cholesterol (HDLC) were 1.00 (95% CI: 0.99, 1.01) and 0.96 (95% CI: 0.94, 0.99), respectively.  Figure 4 (*I*^2^ = 74.9%, *P* = 0.001) and Figure 5 (*I*^2^ = 0%, *P* = 0.770) demonstrated significant interstudy heterogeneity for both analyses. Subgroup analysis showed that TC was not associated with POAG risk in case-control studies and cross-sectional studies. In addition, HDLC was negatively correlated with the risk of POAG with an OR of 0.96 (95% CI: 0.94, 0.99), but not in the cross-sectional study (OR = 0.86, 95% CI: 0.63, 1.16). No publication bias was found, *P* = 0.707 and *P* = 0.221 by Begg's test, respectively.

## 4. Discussion

Abundant prior studies have reported the relationship between abnormal lipid level and risk of POAG with no consistent conclusions. Abnormal lipid metabolism usually refers to the increase of TC or TG, which may be accompanied by the decrease of HDLC [27]. Lipid metabolism is an important risk factor for cardiovascular disease [270]. Previous studies have suggested that abnormal lipid metabolism may increase POAG risk by reducing blood flow velocity and changing lipid components in the aqueous humour [24, 25]. Apolipoprotein B and apolipoprotein E in the aqueous humour of POAG patients were significantly increased, which may then change cholesterol transport. On the other hand, genetic factors may also modulate the the association between lipid metabolism and risk of POAG. It has been shown that several genes involved in lipid metabolism were significantly related to POAG risk, such as ATP binding cassette subfamily A member 1gene and caveolin 1 gene [28, 29].

The aim of this study is to assess the correlation between lipid abnormalities or specific blood lipid levels and the risk of POAG. The results showed that dyslipidemia increased the risk of POAG in cross-sectional and case-control studies, but not in cohort studies. This disparity might be related to the effect of confounding factors. For instance, patients with diagnosed hyperlipidemia were more likely to receive treatment. In a large cohort study by Newman-Casey et al., significant negative correlation between hyperlipidemia and POAG (hazard ratio 0.95, 95% CI 0.91, 0.98) was noted even after correcting for a series of covariates. In previous studies, statins have been shown to reduce the adverse effect of hyperlipidemia on POAG by decreasing intraocular pressure while reducing cholesterol [30]. It is currently unclear whether hyperlipidemia and the use of lipid-lowering medications are independent risk factors for POAG, which should be further clarified in future studies.

For a specific blood lipid parameter, this study did not find that TC and TG were related to an increased risk of POAG. A recent cross-sectional study in Singapore found that high levels of HDL-3 cholesterol were associated with a reduced risk of POAG (OR, 0.91; 95% CI, 0.84-0.99, *P* = 0.021), but no association was found between conventional lipids (e.g., TC, HDLC, and low-density lipoprotein cholesterol) and POAG [31]. Although we noted a negative correlation between HDLC and POAG, it is limited by small sample size and needs further confirmation.

This study suffers from several limitations. First, significant interstudy heterogeneity was noted, which might be related to the diversity of study designs and subject population. However, further subgroup analysis or meta-regression to clarify the source of heterogeneity was difficult due to quantitative limitations. Second, part of the results of this study included less than 10 studies. The funnel plot and asymmetry test methods cannot judge whether the funnel is symmetrical or not due to the low-test performance. More original research is needed in the future to clarify our preliminary conclusions. Third, most of the studies included were cross-sectional or case-control in design, and the number of cohort studies was small, so the causal relationship between dyslipidemia and POAG risk could not be inferred. Fourth, this study failed to clarify the impact of other identified risk factors for POAG. In addition, the OR values reported in some studies were not corrected by covariates, especially intraocular pressure. Finally, this study analyzed the correlations between specific lipid and risk of POAG, providing evidence to clarify the impact of individual lipid on the risk of POAG. However, given the small number of studies included, additional studies are needed in the future to firmly establish this conclusion.

## Figures and Tables

**Table 1:** The basic information of reported studies on the risk of dyslipidemia.

Author	Year	Research type	Data sources	OAG diagnostic criteria	Diagnostic criteria of dyslipidemia	Inclusion period	Age
Jung et al. [11]	2020	Cohort	KNHIS-NSC 2002-2013	Complied with ICD-10 H40.1 and received the prescription of anti-glaucoma drugs during the study period	Hypercholesterolemia: Conformed to ICD-10 E78; received cholesterol drug prescription or TC ≥ 240 mg/dL	2002-2008	≥65 years old, accounting for 16.2%
Rim et al. [12]	2018	Case-control	KNHIS-NSC 2002-2013	Complied with KCD H401 and received the prescription of antiglaucoma drugs during the study period	Hyperlipidemia: accorded to KCD classification	2004-2007	Middle aged and elderly people
Lee et al. [13]	2017	Cross-sectional	KNHANES 2008-2012	Complied with MISGEO-K	Hyperlipidemia: received cholesterol drug prescription or TC ≥ 240 mg/dL	2008-2012	>40 years old
Chen et al. [14]	2016	Case-control	NHI	Complied with ICD-9-CM 365.11	Hyperlipidemia: ICD-9-CM 272	2001-2011	>40 years old, with an average of 57 years old
Kim et al. [15]	2016	Cross-sectional	KNHANES 2010-2012	Complied with ISGEO	Hyperlipidemia: TG ≥ 150 mg/dL or cholesterol drug treatment	2008-2012	>40 years old, with an average of 56 years old
Chung et al. [16]	2014	Cross-sectional	the Longitudinal Health Insurance Database 2000 (LHID2000) of NHI	Complied with ICD-9-CM 365.1 or 365.11	Not mentioned	2002 -2012	≥18 years old
Newman-Casey et al. [17]	2011	Cohort	US i3 InVision data Mart database	ICD-9-CM 365.1, 365.10, 365.11, 365.12 and 365.15	Hyperlipidemia: ICD-9-CM	2001-2007	>40 years old
Lin et al. [18]	2010	Case-control	NHI	ICD-9-CM 365.1-365.11	Elixhauser comorbidity index [19]	2005	>40 years old
Motsko and Jones [20]	2008	Case-control	US Ingenix LabRx database	ICD-9-CM 365.1	Lipid metabolism disorder: ICD-9-CM 272	2001-2004	With an average of 73.6 years old
Girkin et al. [21]	2004	Cohort	The Birmingham (Alabama) Department of Veterans Affairs Medical Center (BVAMC)	ICD-9-CM 365.1	Lipid metabolism disorder: ICD-9-CM 272	1997-2002	>50 years old
Kim et al. [5]	2016	Cross-sectional	KNHANES 2008-2011	MISGEOCK I or II standard	Disorder of lipid metabolism: TC > 200 mg/dL or LDLC > 130 mg/dL or HDLC < 60 mg/dL or TG > 150 mg/dL	2008-2011	>40 years old, with an average age of 59.7 years old

Note: The Korean National Health Insurance System-National Sample Cohort (KNHIS-NSC), Korean National Health and Nutrition Examination Survey (KNHANES)， the International Classification of Diseases, 10th Revision (ICD-10), Korean Classification of Diseases (KCD), the Modified International Society of Geographical and Epidemiological Ophthalmology Criteria for the Korean Population (MISGEO-K), the Modified International Society of Geographical and Epidemiological Ophthalmology Criteria (MISGEO), the International Classification of Diseases, 9th Revision, Clinical Modification (ICD-9-CM), Taiwan National Health Insurance plan (NHI), and the International Society of Geographical and Epidemiological Ophthalmology Criteria (ISGEOC).

**Table 2:** The basic situation of reported studies on blood lipid level and disease risk.

Authors	Year	Research type	Data sources	OAG diagnostic criteria	Report blood lipid parameters	Blood lipid unit	Inclusion period	Age
Lei et al.[22]	2020	Case-control	Collected by Department of Ophthalmology and Visual Sciences; eye, ear, nose, and throat, Hospital of Fudan University	Complied with ISGEOC standards	TG, TC	Per mmol/L	2017	Average age of 60 years old
Shon and Sung [23]	2019	Cross-sectional	KNHANES 2018-2020	Complied with ISGEOC standards	TG, TC, HDLC	Per SD	2008-2012	Average age of 63 years old
Wu [24]	2019	Case-control	Shantou University -Chinese University of Hong Kong joint Shantou international eye center	POAG includes HTG and NTG, which need to meet the inclusion criteria, respectively	TG, TC, HDLC, LDLC	Per mmol/L	——	>40 years old
Tang et al.[25]	2017	Case-control	Eye, ear, nose, and throat, Hospital of Fudan University	Not mentioned	TC, HDLC	Per mmol/L	2015-2016	Average age of 40 years old
Kim, et al.[26]	2014	Cross-sectional	KNHANES 2009–2010	Complied with ISGEOC standards	TG, TC, HDLC, LDLC	Per mg/dL	2009–2010	19-39 years old

Note: Korean National Health and Nutrition Examination Survey (KNHANES), the Modified International Society of Geographical and Epidemiological Ophthalmology Criteria (ISGEO), the Modified International Society of Geographical and Epidemiological Ophthalmology Criteria for the Korean Population (MISGEO-K), total cholesterol (TC), total triglyceride (TG), high density lipoprotein cholesterol (HDL-C), and low density lipoprotein cholesterol (LDL-C).

**Table 3:** Evaluation scores of included studies using the Viswanathan M design scale.

	1	2	3	4	5	6	7	8	9	10	11	12	13	14	15	总分
Jung et al. [11]	1	1	1	1	1	1	0	0	1	0	1	0	1	0	1	10
Rim et al. [12]	1	1	1	1	1	1	0	0	1	0	1	0	1	0	1	10
Lee et al. [13]	1	1	1	1	1	1	0	0	1	0	1	0	1	0	1	10
Chen et al. [14]	1	1	1	1	1	1	0	0	1	0	1	0	1	1	1	11
Kim et al. [15]	1	1	1	1	1	1	0	0	1	0	1	0	1	0	1	10
Chung et al. [16]	1	1	0	0	0	1	0	0	0	0	1	0	1	0	1	6
Newman-Casey et al. [17]	1	1	1	1	1	1	0	0	1	0	1	0	1	0	1	10
Lin et al. [18]	1	1	0	0	0	1	0	0	0	0	1	0	1	0	1	6
Motsko and Jones. [20]	1	1	0	0	0	1	0	0	1	0	1	0	1	0	1	7
Girkin et al. [21]	1	1	1	0	1	1	0	0	1	0	1	0	1	0	1	9
Kim et al. [5]	1	1	1	1	1	1	0	0	1	0	1	0	1	0	1	10
Lei et al. [22]	1	1	1	1	1	1	0	0	1	0	1	0	1	0	1	10
Shon and Sung [23]	1	1	1	1	1	1	0	0	1	0	1	0	1	0	1	10
Wu et al. [24]	1	1	0	1	1	1	0	0	1	0	1	0	1	0	1	9
Tang et al. [25]	1	1	0	1	1	1	0	0	1	0	1	0	1	0	1	9

Note: Quality criteria and evaluation of design and data analysis for observational studies criteria (1) Was the research question or objective in this paper clearly stated? (2) Was the study population clearly specified and defined? (3) Was the study population representative of the general population? (4) Was the participation rate of eligible persons at least 50%? (5) Were all the subjects selected or recruited from the same or similar populations (including the same time period)? Were inclusion and exclusion criteria for being in the study prespecified and applied uniformly to all participants? (6) Were sample size justification, power description, or variance and effect estimates provided? (7) For the analyses in this paper, were the exposures of interest measured prior to the outcomes being measured? (8) Was the time frame sufficient so that one could reasonably expect to see an association between exposure and outcome if it existed? (9) Were the exposure measures (independent variables) clearly defined, objective, valid, reliable, and implemented consistently across all study participants? (10) Were the exposures assessed more than once over time? (11) Were the outcome measures (dependent variables) clearly defined, valid, reliable, and implemented consistently across all study participants? (12) Were the outcome assessors blinded to the exposure status of participants? (13) Was the statistical analysis appropriate? (14) Was loss to follow-up after baseline 20% or less? (15) Were the key potential confounding variables measured and adjusted statistically for their impact on the relationship between exposures and outcomes? (1: yes; 0: no or not applicable).

## Data Availability

The data used to support the findings of this study are available from the corresponding author upon request.

## Supplementary Materials

Supplementary Materials This section provides additional information about the funnel chart for reporting the relationship between dyslipidemia and the risk of POAG (Supplementary Figure S1).
