# Supplementary figures and images for: Meta-Analysis of Dyslipidemia and Blood Lipid Parameters on the Risk of Primary Open-Angle Glaucoma
Source: Comput Math Methods Med. 2022 Sep 21;2022:1122994. doi: 10.1155/2022/1122994 (PMC9519322; doi:10.1155/2022/1122994)

Funnel plot with subgroups

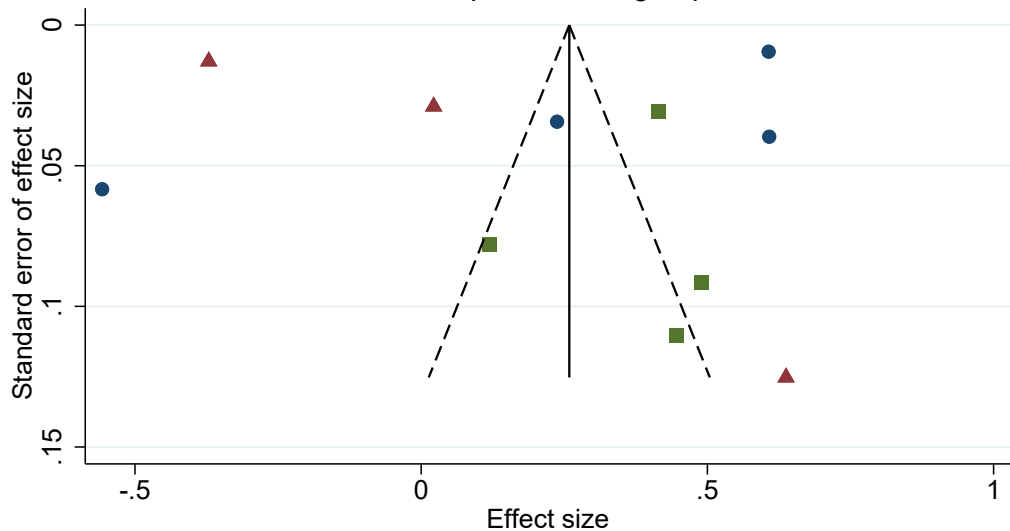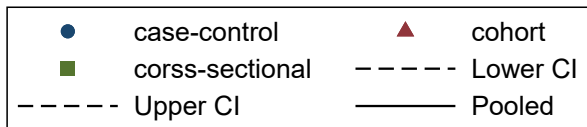

Supplement: Supplementary Materials — This section provides additional information about the funnel chart for reporting the relationship between dyslipidemia and the risk of POAG (Supplementary Figure S1). [file 1122994.f1.zip › editable Supplementary Figure S1.pdf]
